# The biomechanical effects of insoles with different cushioning on the knee joints of people with different body mass index grades

**DOI:** 10.3389/fbioe.2023.1241171

**Published:** 2023-09-15

**Authors:** Rui Jia, Fei Wang, Jiang Jiang, Hongtao Zhang, Jianyi Li

**Affiliations:** ^1^ Guangdong Provincial Key Laboratory of Digital Medicine and Biomechanics, Guangdong Engineering Research Center for Translation of Medical 3D Printing Application, Department of Anatomy, School of Basic Medical Sciences, Southern Medical University, Guangzhou, China; ^2^ Zhongshan Torch Development Zone People’s Hospital, Zhongshan, China

**Keywords:** obesity, knee, insoles, cushioning, biomechanics

## Abstract

**Background:** Enhancing knee protection for individuals who are overweight and obese is crucial. Cushioning insoles may improve knee biomechanics and play a significant protective role. However, the impact of insoles with varying cushioning properties on knee joints in individuals with different body mass index (BMI) categories remains unknown. Our aim was to investigate the biomechanical effects of insoles with different cushioning properties on knee joints across different BMI grades.

**Methods:** Gravity-driven impact tests were used to characterize the cushioning properties of three types of Artificial Cartilage Foam (ACF18, 28, and 38) and ethylene-vinyl acetate (EVA) insoles. Knee joint sagittal, coronal, and vertical axis angles and moments were collected from healthy-weight (BMI 18.5–23.9 kg/m^2^, *n* = 15), overweight (BMI 24.0–27.9 kg/m^2^, *n* = 16), and obese (BMI ≥28.0 kg/m^2^, *n* = 15) individuals randomly assigned four different insoles during a drop jump. The Kruskal–Wallis test and mixed model repeated measures analysis of variance were used to compare differences among cushioning and biomechanical data across various insoles, respectively.

**Results:** ACF showed higher cushioning than EVA, and ACF38 was the highest among the three types of ACF (all *p* < 0.001). During the drop jump, the knee flexion angles and moments of the ACF insoles were lower than those of the EVA insoles, the knee adduction angles of the ACF18 and ACF28 insoles were lower than those of the EVA insoles, and ACF18 insoles increased the first cushion time (all *p* < 0.05) for all participants in whom biomechanical variables demonstrated no interactions between insoles and BMI. Regarding the BMI-dependent biomechanical variables, compared with the EVA insoles, ACF28 insoles decreased the knee flexion angle and ACF38 insoles decreased the knee adduction and rotation moment in the healthy-weight group; ACF18 insoles decreased the knee flexion angle and ACF38 insoles decreased the knee moment in the overweight group; ACF28 insoles decreased the knee flexion and adduction moment, and ACF38 insoles decreased the knee flexion angle and rotation moment in the obese group (all *p* < 0.05).

**Conclusion:** Insoles with higher cushioning properties could improve knee biomechanics and provide better knee joint protection in people across different BMI ranges.

## 1 Introduction

The worldwide prevalence of obesity has become a major health concern (Heymsfield and Wadden, 2017). Obesity has been identified as a significant risk factor for musculoskeletal disorders, particularly knee osteoarthritis (Jiang et al., 2021). Increased body mass may produce biomechanical alterations of the knee joint and augment the risk of knee injury with repetitive loading during weightbearing activities (Kanthawang et al., 2021). Sibella et al., 2003 found that obese individuals showed higher knee joint torque and angle during the sit-to-stand motion, leading to abnormal changes in the contact position of the articular cartilage and increased articular cartilage wear and knee joint swelling (Runhaar et al., 2011). Obese adults also demonstrated a higher knee adduction moment which is associated with knee pain and function loss (Pereira et al., 2021). Abnormal knee angle and moment may ultimately favor the development of osteoarthritis over time, since any minor alteration in the kinematics or kinetics of the joint can lead to a clinically relevant change in the musculoskeletal system (Capodaglio et al., 2021). Therefore, it is important to enhance knee protection for obese people and delay the onset and progression of knee osteoarthritis.

Using the lateral wedge insole is a conservative management strategy for knee osteoarthritis, which can improve femorotibial angle to reduce pain and optimize function (Rodriguez-Merchan and De La Corte-Rodriguez, 1995; Zhang et al., 2018). The wedged insoles only involved the structure factor of insoles. As another material factor, the cushioning property is also important. In theory, reducing the vertical impact force of the ground may decrease the load to protect the knee joints, however, the effect of cushioning insoles on knee protection is controversial. Turpin et al., 2012 reported that cushioning insoles significantly reduce physical dysfunction in patients with knee osteoarthritis. Lewinson and Stefanyshyn, 2019 also found that cushioning insoles could improve biomechanical indicators such as knee abduction angular impulses to relieve knee pain for runners. In contrast, a randomized controlled trial showed that cushioning shoes might not decrease the injury risk in overweight runners (Malisoux et al., 2020). To address this controversy, we hypothesized that the different cushioning properties of the insoles used in published studies may have affected the results; few studies have explored the effects of cushioning insoles on knee joint protection in individuals with different BMI grades. Confirming the effect of cushioning insoles and choosing the appropriate insole cushioning property for overweight and obese people may prevent early knee abnormalities during exercise.

The cushioning property of insole materials is considered to be key for knee protection (Kermen and Mohammadi, 2021). Ethylene-vinyl acetate (EVA) foam is commonly used as the traditional cushioning material in insole manufacturing (Anggoro et al., 2021). Fu et al., 2022 designed special structures with a heel-to-toe drop of 16 mm using EVA to improve the cushioning effect; however, the stability was uncertain, and maintaining excellent cushioning in long exercises was difficult due to EVA structural collapse (Wang et al., 2012). To enhance the cushioning properties of insoles, Artificial Cartilage Foam (ACF) was discovered. ACF, whose matrix is a molecular structure specially designed viscoelastic polyurethane, is a novel mixed cellular material and a biomimetic metamaterial with a three-dimensional ultrastructure similar to human cartilaginous tissue (Wang B. et al., 2020). Electron and atomic force microscopic scanning showed that the surface of ACF is distributed with connective micron-sized pores with a shape close to circular and nanoscale bumps (Chen S. et al., 2022). It can absorb up to 97.1% of the impact energy, and the energy-absorbing capacity only decreases by 0.4% after five impacts (Chen S. et al., 2022). Its cushioning and energy absorption performance significantly exceeds those of ordinary cushioning materials (Wang B. et al., 2020). However, the biomechanical effects of insoles with higher cushioning properties on the knee joints of people with different body mass index (BMI) grades remains unknown.

This study investigated the effects of insoles with different cushioning properties on the knee joints of people with different BMI grades based on the analysis of different insole cushioning properties and knee kinematic and kinetic variables. We hypothesized that insoles with higher cushioning properties could decrease the knee angle and moment with different BMI grades.

## 2 Materials and methods

### 2.1 Materials

Four different insoles were selected for this study: EVA (hardness of 35 Shore C), ACF18 (hardness of 25 Shore C), ACF28 (hardness of 30 Shore C), and ACF38 (hardness of 35 Shore C). The four insoles are identical, with a heel-to-toe drop of 2 mm, and all are made of flat material. The three types of ACF insoles were named based on their density: ACF18 (0.18 g/cm^3^), ACF28 (0.28 g/cm3), and ACF38 (0.38 g/cm^3^). The density of EVA insoles that were used as the control condition was 0.11 g/cm^3^.

According to ASTM-F1976-13 (American Society for Testing and Materials, 2013), gravity-driven impact tests were used to characterize the cushioning properties of the four insoles at the heel using a material testing machine (Instron, United States). Each insole at the rear-part was subjected to a series of 30 impacts per minute, consisted of a 50-mm free fall of 8.5-kg gravity-driven missile, with a minimal interval of 2.0 s (Malisoux et al., 2023).

### 2.2 Participants

The study included 46 healthy adult participants (21 women and 25 men) aged 20–36 years old who had no lower extremity deformities or injuries within the previous 6 months. Exclusion criteria were neuromuscular, psychological, and/or cardiopulmonary conditions that could significantly affect athletic abilities. All participants were divided into three groups according to the BMI classification criteria of China, including a healthy weight group (HW, BMI of 18.5–23.9 kg/m^2^), overweight group (OW, BMI of 24.0–27.9 kg/m^2^) and obesity group (OB, BMI of 28.0 kg/m^2^ or higher) (Chen et al., 2004). This study was approved by the Ethics Committee (2022–0001). Each participant received a full study description and signed an informed consent form. Sample size calculation was performed using the PASS software (NCSS, United States), based on the Geisser-Greenhouse F-test algorithm. The number of participants required in each group was 14.

### 2.3 Procedures

**FIGURE 1:**
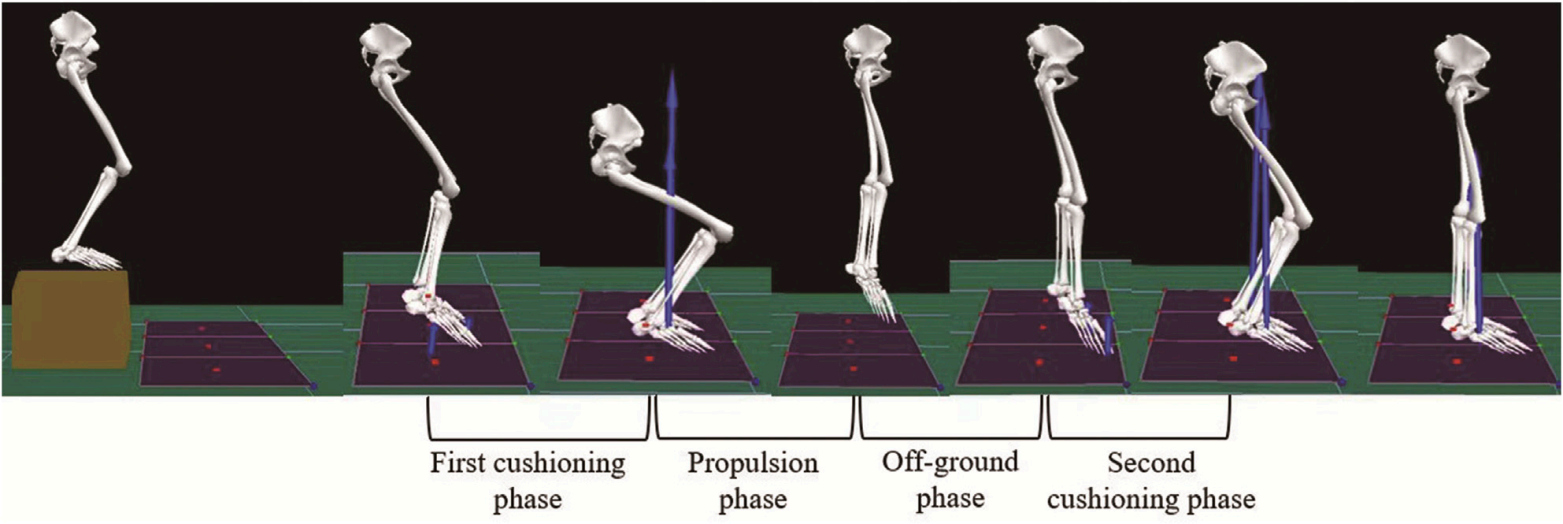
The standardized process of the drop jump (DJ).

To avoid a confounding factor, each participant wore the same type of ordinary sports shoes without special structure on the market, United Kingdom size 5.0–8.0, mass 270–285 g, offset 6 mm, with EVA as the midsole material. Thirty-six reflective marker points were placed bilaterally on the lower extremities. The location of the marker points was chosen based on the CAST lower-body model (Cappozzo et al., 1995). The motion of the markers was captured with infrared motion capture cameras (Qualisys, Sweden) and ground reaction forces were acquired by two force platforms (Kistler, Switzerland), which both collection frequency were 100 Hz. All participants were organized to learn the drop jump (DJ); they watched a standard DJ video, as well as a demonstration by a professional; afterward, they practiced three to five times until the researcher ensured that their DJ met the criteria. The standard DJ involved a jump from the front of a box 30 cm high with arms swinging naturally at the sides of the body, landing with both feet on the center of two separate force plates, and an immediate vertical jump as high as possible (Figure 1). Participants were given a 20 min warm-up and habituated to insoles and shoes. Participants were tested with four insoles in a random order by using a random number generator. In order to ensure that the participants were blinded, a researcher was responsible for inserting the insoles into each shoe. The participants completed the DJ three successful trials with each insole. Participants were given 5 min of recovery at the end of each DJ, and 30 min of recovery guaranteed between each type of insole (Vercruyssen et al., 2016).

### 2.4 Data collection

The DJ was divided into four phases (Figure 1): the first cushioning phase (the time that the participants’ feet touched the force platforms until the time of maximum knee flexion), the propulsion phase (the time from maximum knee flexion until the time that the participants’ feet left the force platforms), off-ground phase (the time that the participants’ feet left the force platforms until the time that their feet touched the force platforms again), and second cushioning phase (the time that the participants’ feet touched the force platforms again until the time of maximum knee flexion). The temporal variables included the time of each phase.

Kinematic and kinetic data were analyzed using Visual 3D analysis software (C-Motion Inc., United States). A fourth-order low-pass Butterworth filter was used with cut-off frequencies of 100 Hz (kinetic) and 10 Hz (kinematic). The variables were the knee joint *X*-*Y*-*Z*-axis angles and moments at the foot contact with the force platforms and the peak vertical ground reaction force during the DJ. The *X*-axis represents the frontal axis (positive direction indicates knee flexion), the *Y*-axis represents the sagittal axis (positive direction indicates knee abduction), and the *Z*-axis represents the vertical axis (positive direction indicates internal knee rotation). Moments were normalized to the participant’s body mass. All biomechanical variables were obtained from the participants’ dominant side. Limb dominance was determined by asking participants which limb they would prefer to kick a ball (Carcia et al., 2019). The average of all three successful trials for any particular insole was used for statistical analysis.

### 2.5 Statistics

Insole cushioning parameters and biomechanical data were analyzed using the statistical package SPSS (SPSS Inc., United States). The Shapiro-Wilk test was used to verify the possible normal distribution of all parameters. Independent samples nonparametric tests were used to compare differences among the four cushioning insole parameters, and the Kruskal–Wallis test was used for pairwise comparisons because these data did not satisfy the normality distribution. Because the biomechanical estimated parameters satisfied the normality distribution, mixed model repeated measures analysis of variance was used to compare the means among the test conditions, followed by multiple statistical comparisons. For all statistical tests, the significance level was set at *p* < 0.05.

## 3 Results

### 3.1 Cushioning properties

The cushioning properties of the four insoles are presented in Table 1. The time to the point where the maximum displacement occurred (Tm) was the same for the four insoles. Differences in maximum displacement (MD) were found among all insoles (*p* < 0.001). The ACF38 (*p* < 0.001) and ACF28 (*p* < 0.001) were less than the EVA, and the ACF38 was less than the ACF18 (*p* < 0.001) for maximum impact acceleration (MIA). The absorption energy (AE) in ACF38 (*p* < 0.001) and ACF28 (*p* < 0.001) was higher than that in EVA, whereas that in ACF38 (*p* = 0.002) and ACF28 (*p* = 0.004) was higher than that in ACF18.

**TABLE 1:** Comparison of the four types of insoles regarding cushioning properties.

	EVA	ACF18	ACF28	ACF38	P
T_m_ (s)	1.03	1.03	1.03	1.03	
MD (mm)	10.43 (10.48–10.42)^*^ ^b^	9.51 ± 0.03^a^	8.74 (8.75–8.68)^*^ ^ab^	6.85 (−6.94-6.84)^*^ ^abc^	<0.001^#^
MIA (mm/s^2^)	493,943.12 (493,682.88–497480.20)^*^	492,831.25 ± 448.78	450,106.93 ± 1280.92^a^	394,925.60 ± 1190.24^ab^	<0.001^#^
AE (J)	1.43 ± 0.006	2.01 (1.96–2.04)^*^	2.33 ± 0.02^ab^	2.33 (2.32–2.45)^*^ ^ab^	<0.001^#^

^a^ , Significant difference among the ACF38, ACF28, ACF18, and EVA insoles.

^b^ , Significant difference among the ACF38, ACF28, EVA, and ACF18 insoles.

c , Significant difference between the ACF38 and ACF28 insoles.

Values other than T_m_ are expressed as mean ± standard deviation except where the data did not satisfy the normality distribution, where these data are presented as median (interquartile range).

^*^, Non-normally distributed data.

T_m_, time to the point where maximum displacement occurred; MD, maximum displacement; MIA, maximum impact acceleration; AE, absorption energy; ACF, artificial cartilage foam; EVA, ethylene-vinyl acetate.

#, *p*-value <0.05.

### 3.2 Participants

Forty-six participants were enrolled in this study, including 15 HW participants, 16 OW participants, and 15 OB participants. All participants were reported to be right-leg-dominant. The participant demographics are shown in Table 2.

**TABLE 2:** Participant information.

	HW (n = 15)	OW (n = 16)	OB (n = 15)	P
Sex (Male/Female)	8/7	8/8	9/6	
Age (Years)	25.94 ± 2.29	23.50 (22.00–26.75)^*^	23.55 ± 2.38	0.114
Height (cm)	164.94 ± 7.08	173.05 ± 10.26^b^	167.73 ± 8.24	0.027^#^
Body mass (kg)	60.22 ± 7.09	79.05 ± 9.96^b^	83.00 (78.50–92.00)^*^ ^a^	<0.001^#^
BMI (kg/m^2^)	22.06 ± 1.37	26.60 (25.53–27.18)^*^ ^b^	30.90 ± 2.77^ac^	<0.001^#^

^a^ , Significant difference between the OB and HW groups.

^b^ , Significant difference between the OW and HW groups.

c , Significant difference between the OB and OW groups.

Values other than sex are expressed as mean ± standard deviation, except where the data are non-normally distributed, where these data are presented as median (interquartile range).

^*^, Non-normally distributed data.

^#^, *p*-value <0.05. BMI, body mass index; HW, healthy weight; OB, obese; OW, overweight.

### 3.3 Biomechanical outcomes

The biomechanical variables that demonstrated no interactions between insoles and BMI are presented in Table 3. The knee flexion angle at the time of peak vertical ground reaction force during the second jump for the ACF18 (*p* = 0.025), ACF28 (*p* = 0.014) and ACF38 (*p* = 0.002) insoles was lower than that for the EVA insoles. The knee adduction angle at the second contact in the ACF18 insoles and ACF28 insoles was lower than that in the EVA and ACF38 insoles (all *p* < 0.05). The knee flexion moment at the time of peak vertical ground reaction force during the first jump for the ACF38 was lower than that for the ACF18 insoles (*p* = 0.007). Regarding the knee flexion moment at the time of peak vertical ground reaction force during the second jump, significant differences were observed between EVA and ACF28 insoles (*p* = 0.001), ACF18 and ACF28 insoles (*p* = 0.001), as well as ACF38 and ACF28 insoles (*p* < 0.001). The first cushion time in the ACF18 insoles was longer than that in the ACF38 (*p* = 0.006), ACF28 (*p* = 0.019), and EVA (*p* = 0.003) insoles.

**TABLE 3:** Descriptive statistics for the biomechanical variables that do not demonstrate an interaction between insoles and body mass index in four insole conditions.

	EVA	ACF18	ACF28	ACF38	P (interaction)	P
Knee joint motion (°)						
Flexion angle at peak VGRF2	51.54 ± 8.20	48.58 ± 9.76^a^	48.60 ± 11.14^a^	48.40 ± 8.64^a^	0.456	0.002^#^
Adduction angle at second contact	−3.36 ± 3.57	−2.52 ± 3.45^a^	−2.54 ± 3.51^a^	−3.18 ± 3.55^bc^	0.281	0.008^#^
Flexion moment at peak VGRF1	1.55 ± 0.69	1.70 ± 0.56^a^	1.59 ± 0.54	1.48 ± 0.55^b^	0.764	0.034^#^
Flexion moment at peak VGRF2	1.28 ± 0.48	1.31 ± 0.48	1.57 ± 0.47^ab^	1.22 ± 0.25^c^	0.090	<0.001^#^
First cushion time (s)	0.27 ± 0.09	0.35 ± 0.18^a^	0.29 ± 0.10^b^	0.27 ± 0.09^b^	0.058	0.006^#^

^#^, *p*-value <0.05.

^a^ , Significant difference among the ACF38, ACF28, ACF18, and EVA groups.

^b^ , Significant difference among the ACF38, ACF28, and ACF18 groups.

^c^ , Significant difference among the ACF38 and ACF28 groups.

Values are expressed as mean ± SD.

ACF, artificial cartilage foam; EVA, ethylene-vinyl acetate; Peak VGRF1, peak vertical ground reaction force during the first jump; Peak VGRF2, peak vertical ground reaction force during the second jump.

The biomechanical variables that demonstrated an interaction between insoles and BMI are presented in different groups based on BMI (Table 4). In the HW group, the knee flexion angle at the second contact was significantly lower for the ACF28 insoles compared with the EVA insoles (*p* = 0.046). The knee adduction moment at initial contact for the ACF38 insoles was lower than that for the EVA insoles (*p* < 0.001). The knee rotation moment at the time of peak vertical ground reaction force during the second jump for the ACF18, ACF28, and ACF38 insoles was lower than that for the EVA insoles (all *p* < 0.05).

**TABLE 4:** Descriptive statistics for the biomechanical variables that demonstrates an interaction between insoles and body mass index in four insole conditions of the healthy weight, overweight, and obesity groups.

	EVA	ACF18	ACF28	ACF38	P (interaction)	P
HW						
Flexion angle at second contact (°)	26.94 ± 6.35	25.09 ± 6.54	24.67 ± 5.36^a^	26.14 ± 8.16	0.001^#^	<0.001^#^
Adduction moment at initial contact (Nm/kg)	−0.10 ± 0.05	−0.08 ± 0.07	−0.07 ± 0.11	−0.04 ± 0.03^a^	0.017^#^	<0.001^#^
Rotation moment at peak VGRF2 (Nm/kg)	−0.17 ± 0.09	−0.12 ± 0.11 ^a^	−0.12 ± 0.09^a^	−0.12 ± 0.10^a^	0.019^#^	0.017^#^
OW						
Flexion angle at second contact (°)	27.73 ± 11.02	23.07 ± 9.47^a^	26.45 ± 8.24	24.55 ± 5.86	0.001^#^	<0.001^#^
Flexion moment at second contact (Nm/kg)	0.04 ± 0.11	−0.05 ± 0.13	0.03 ± 0.14	−0.0001 ± 0.14^a^	0.006^#^	0.024^#^
Adduction moment at initial contact (Nm/kg)	−0.10 ± 0.07	−0.05 ± 0.06^a^	−0.04 ± 0.13	−0.01 ± 0.06^a^ ^b^	0.017^#^	<0.001^#^
Rotation moment at second contact (Nm/kg)	−0.02 ± 0.01	−0.01 ± 0.01	−0.01 ± 0.01	−0.005 ± 0.02^a^	0.001^#^	0.025^#^
OB						
Flexion angle at second contact (°)	26.95 ± 5.41	25.38 ± 6.56	21.75 ± 6.93^a^ ^b^	20.12 ± 3.29^a^ ^b^	0.001^#^	<0.001^#^
Flexion moment at second contact (Nm/kg)	−0.06 ± 0.07	0.04 ± 0.13	−0.001 ± 0.12^a^	0.02 ± 0.19	0.006^#^	0.017^#^
Adduction moment at initial contact (Nm/kg)	−0.11 ± 0.09	−0.07 ± 0.10	−0.03 ± 0.03^a^	−0.09 ± 0.12	0.017^#^	<0.001^#^
Rotation moment at peak VGRF2 (Nm/kg)	−0.12 ± 0.18	−0.06 ± 0.09	−0.05 ± 0.11	−0.02 ± 0.10^a^ ^b^	0.019^#^	0.023^#^

^#^, *p*-value <0.05.

^a^ , Significant difference among the ACF38, ACF28, ACF18, and EVA groups.

Values are expressed as mean ± SD.

ACF, artificial cartilage foam; EVA, ethylene-vinyl acetate; Peak VGRF1, peak vertical ground reaction force during the first jump; Peak VGRF2, peak vertical ground reaction force during the second jump.

In the OW group, the knee flexion angle at the second contact in the ACF18 insoles was lower than that in the EVA insoles (*p* = 0.017). The knee flexion moment at the second contact for the ACF38 insoles was lower than that for the EVA insoles (*p* = 0.024). The knee adduction moment at initial contact for the ACF38 insoles and ACF18 insoles was lower than that in the EVA insoles (all *p* < 0.05); Furthermore, the same value for the ACF38 insoles was lower than that for the ACF18 insoles (*p* < 0.05). Regarding the knee rotation moment at the second contact, the ACF38 insoles and the EVA insoles were significantly different (*p* = 0.025).

In the OB group, the knee flexion angle at the second contact in the ACF28 and ACF38 insoles was lower than that in the EVA and ACF18 insoles (all *p* < 0.05). The knee flexion moment at the second contact for the ACF28 insoles was lower than that for the EVA insoles (*p* = 0.017). The knee adduction moment at the initial contact for the ACF28 insoles was lower than that for the EVA insoles (*p* = 0.001). The knee rotation moment at the time of peak vertical ground reaction force during the second jump for the ACF38 insoles (all *p* < 0.05) was lower than that for the EVA and ACF18 insoles.

## 4 Discussion

This study investigated the effects of insoles with different cushioning properties on the knee joint in people with different BMI grades based on insole cushioning properties and knee biomechanical variable analysis. Insoles with higher cushioning properties could significantly decrease the angle and moment of knee flexion and adduction, as well as the moment of rotation; while prolonging the first cushioning time of the DJ.

Cushioning insoles should provide adequate cushioning while creating a safe and stable mechanical environment for the lower extremities (Withnall et al., 2006). They should rapidly absorb energy and sustain low deformation when the lower extremities are subjected to large impacts (Yang et al., 2022). This study used a gravity-driven impact test to quantify the energy absorption and deformation levels of three types of ACF and EVA materials. The absorption energy in the ACF38 and ACF28 insoles was greater than that in the ACF18 and EVA insoles, whereas the maximum displacement differences were found between all insoles (ACF38<ACF28<ACF18<EVA). This confirmed that all ACF insoles had higher cushioning properties than EVA insoles when the density of EVA insoles was lower than those of ACF insoles in this study. With the increase of density and hardness, the cushioning performance of ACF improves accordingly, and the ACF38 insoles were the highest cushioning among the three types of ACF insoles.

Some studies have explored the role of protective aids. Malisoux et al. (Malisoux et al., 2023) and Vercruyssen et al., 2016 studied the effects of cushioning shoes on reducing lower extremity injuries by running on a treadmill. However, the results may be affected as running on a treadmill is different from ground running (Riley et al., 2008). Ewing et al. (Ewing et al., 2016) investigated changes in lower-limb muscle function with prophylactic knee bracing during double-leg drop landing from heights of 0.30 m and 0.60 m; however, they found it difficult to evaluate the reactivation level of the neuromuscular system. A DJ was used to screen for abnormal movement patterns to identify the risk of knee injury (Kotsifaki et al., 2022). Not only does a DJ result in a large impact and load of seven times the body weight in the lower extremities, but it is also a pre-programmed motor control landing movement as the vertical jump is performed immediately after landing from a height with anticipatory pre-activation of the lower extremity muscles (Wang et al., 2017; Wang et al., 2021). We chose DJ to investigate the effects of insoles with different cushioning properties on the knee joint in people with different BMI grades.

Increscent knee moment in the sagittal and frontal plane during the DJ may lead to excessive tibia movement and increase the risk of knee injury (Wilder et al., 2021). Some studies have reported lower knee moment in highly cushioned shoes during landing tasks (Xu et al., 2021; Malisoux et al., 2023). Similarly, lower knee flexion, adduction, and rotation moment were also observed in three types of ACF insoles than in EVA insoles for people with different BMI grades, suggesting that insoles with higher cushioning properties can dissipate these loads on the knee and reduce the imbalance of stress on the knee cartilage (Hewett et al., 2015). We compared knee angles among four insoles with different cushioning properties and found that the knee flexion and adduction angles in ACF18, ACF28, and ACF38 insoles were lower due to increased energy dissipation at the foot-shoe-ground interfaces, which could reduce leg compression and center of mass descent during the cushioning phase in higher cushioning insoles (Kulmala et al., 2018). As individuals with knee osteoarthritis present altered sagittal and frontal plane knee angles (Sonoo et al., 2019), ACF insoles may result in improved movement patterns which could decrease the incidence of knee osteoarthritis; however, further research is required. Further, the first cushion time of ACF18 insoles was longer, generally consistent with the result of *in vitro* experiments, which state that higher cushioning is characterized by a longer cushion time (Shorten and Mientjes, 2011). Cushioning insoles were more effective for knee joint protection because a longer cushioning phase may extend the time of impact velocity change applied to the lower extremities and decrease the loading rate for the knee to provide more time to maintain the stability of knee joints during the phase of foot strike (Wang I. et al., 2020). Knee-related injuries are linked to a high vertical ground reaction force during foot striking (Malisoux et al., 2022). However, no difference in the peak vertical ground reaction force was observed among the four insoles in this study, consistent with Fu et al., 2013. This is further supported by Zhang et al., 2005, which showed that the peak impact force substantially increased with increasing effective mass and landing velocity but was relatively insensitive to changes in insole cushioning.

All three types of ACF insoles, which had higher cushioning properties, decreased certain knee biomechanical variables in people with different BMI grades. However, it is still unclear what kind of insole was the most suitable for protecting knee joints in healthy-weight, overweight, or obese people. Higher cushioning properties do not necessarily translate to lower risks of injury in people across different ranges of BMI (Malisoux et al., 2016). There were no quantitative thresholds used to determine “risky” kinematic and kinetic measurements in people with different BMI grades (Agresta et al., 2022) while some studies demonstrated the abnormal motion pattern by a qualitative description, such as a greater knee angle or vertical loading rate. Further research is required to determine the appropriate kinematics and kinetics to offer suitable cushioning insoles to people with different BMI grades and to prevent knee injuries.

### 4.1 Study Limitations

Firstly, we only compared the cushioning properties of three types of ACF insoles and one of EVA insoles with different density. The cushioning properties may be different if the EVA of higher density was chosen to compare with the ACF insoles. How density affects the cushioning property of insoles of different materials need further study. Secondly, some confounding factors, such as experimental shoes, were not considered due to the non-paramedical data sample, which limited the interpretation of the results. Thirdly, the ages of the adult participants are 24–28 years; therefore, caution is necessary when extrapolating these results to all ages. Furthermore, this study did not analyze muscle reactivation using surface electromyography (sEMG) during DJ. The reactivation level of the neuromuscular system is important for knee joint stability. Musculoskeletal modeling and sEMG should be involved in exploring the mechanism of knee joint protection in future research. Finally, this study was cross-sectional and only considered the immediate cushioning effect of the insoles. A long-term and stable cushion is also the key to the protective effect of insoles. Future research should prospectively evaluate the effect of knee prevention with higher cushioning insoles in individuals with different BMI grades during endurance sports.

## 5 Conclusion

During the DJ, insoles with higher cushioning properties significantly decreased knee angle and moment and extended cushion time compared. Therefore, insoles with higher cushioning properties change knee biomechanics, which might provide better protection for people’s knee joints across different BMI ranges. Future research should explore the effect of changes in insole cushioning properties on the knee joint’s internal stress and evaluate the relationship between insole cushioning and knee injuries more directly using musculoskeletal finite element analysis as one of the motion simulations (Chen W. et al., 2022; Song et al., 2023).

## Data Availability

The original contributions presented in the study are included in the article/Supplementary Material, further inquiries can be directed to the corresponding authors.
