# Supplementary material for: The biomechanical effects of insoles with different cushioning on the knee joints of people with different body mass index grades
Source: Front Bioeng Biotechnol. 2023 Sep 15;11:1241171. doi: 10.3389/fbioe.2023.1241171 (PMC10540770; doi:10.3389/fbioe.2023.1241171)
Supplement: Supplementary file 2 [file Table1.DOCX]

**Suppl. Table** Descriptive statistics for the biomechanical variables that do not demonstrate an interaction between insoles and body mass index in four insole conditions

|  | EVA | ACF18 | ACF28 | ACF38 | P （interaction） | P | |
| --- | --- | --- | --- | --- | --- | --- | --- |
| Knee joint motion (°) |  |  |  |  |  |  |  |
| Flexion angle at initial contact | 31.26 ± 7.15 | 29.57 ± 6.74 | 30.95 ± 6.57 | 30.48 ± 6.34 | 0.178 | 0.189 |  |
| Flexion angle at peak VGRF1 | 56.86 ± 12.70 | 54.73 ± 10.41 | 56.52 ± 10.95 | 54.80 ± 9.03 | 0.716 | 0.114 |  |
| Flexion angle at peak VGRF2 | 51.54 ± 8.20 | 48.58 ± 9.76 ^A^ | 48.60 ± 11.14 ^A^ | 48.40 ± 8.64 ^A^ | 0.456 | 0.002 ^#^ |  |
| Adduction angle at initial contact | -2.30 ± 3.70 | -1.56 ± 3.42 | -1.80 ± 3.48 | -1.77 ± 3.83 | 0.161 | 0.065 |  |
| Adduction angle at peak VGRF1 | -2.94 ± 5.15 | -1.98 ± 4.90 | -2.65 ± 5.23 | -2.55 ± 5.26 | 0.756 | 0.064 |  |
| Adduction angle at second contact | -3.36 ± 3.57 | -2.52 ± 3.45 ^A^ | -2.54 ± 3.51 ^A^ | -3.18 ± 3.55 ^B C^ | 0.281 | 0.008 ^#^ |  |
| Adduction angle at peak VGRF2 | -3.00 ± 5.25 | -2.36 ± 5.26 | -2.44 ± 4.75 | -2.47 ± 5.13 | 0.359 | 0.383 |  |
| Rotation angle at initial contact | -3.84 ± 5.47 | -3.66 ± 5.73 | -3.37 ± 5.67 | -3.02 ± 5.98 | 0.348 | 0.422 |  |
| Rotation angle at peak VGRF1 | 0.34 ± 4.73 | -0.21 ± 4.50 | 0.34 ± 5.44 | -0.31 ± 5.90 | 0.177 | 0.214 |  |
| Rotation angle at second contact | -4.23 ± 6.19 | -3.59 ± 6.03 | -3.71 ± 5.78 | -4.04 ± 6.16 | 0.240 | 0.457 |  |
| Rotation angle at peak VGRF2 | -0.77 ± 4.78 | -0.37 ± 5.26 | -0.01 ± 5.80 | -0.97 ± 5.27 | 0.107 | 0.437 |  |
| Knee joint moment (Nm/kg) |  |  |  |  |  |  |  |
| Flexion moment at initial contact | -0.06 ± 0.22 | -0.04 ± 0.26 | -0.03 ± 0.21 | -0.02 ± 0.21 | 0.375 | 0.193 |  |
| Flexion moment at peak VGRF1 | 1.55 ± 0.69 | 1.70 ± 0.56 ^A^ | 1.59 ± 0.54 | 1.48 ± 0.55 ^B^ | 0.764 | 0.034 ^#^ |  |
| Flexion moment at peak VGRF2 | 1.28 ± 0.48 | 1.31 ± 0.48 | 1.57 ± 0.47 ^A B^ | 1.22 ± 0.25 ^C^ | 0.090 | <0.001^#^ |  |
| Adduction moment at peak VGRF1 | -0.24 ± 0.23 | -0.25 ± 0.26 | -0.21 ± 0.26 | -0.23 ± 0.27 | 0.865 | 0.383 |  |
| Adduction moment at second contact | -0.06 ± 0.05 | -0.03 ± 0.15 | -0.05 ± 0.04 | -0.05 ± 0.05 | 0.803 | 0.609 |  |
| Adduction moment at peak VGRF2 | -0.22 ± 0.21 | -0.22 ± 0.24 | -0.20 ± 0.22 | -0.19 ±0.22 | 0.489 | 0.460 |  |
| Rotation moment at initial contact | -0.30 ± 0.04 | -0.03 ± 0.03 | -0.03 ± 0.04 | -0.04 ± 0.03 | 0.076 | 0.466 |  |
| Rotation moment at peak VGRF1 | -0.14 ± 0.17 | -0.14 ± 0.21 | -0.13 ± 0.20 | -0.14 ± 0.20 | 0.505 | 0.925 |  |
| Peak VGRF1 (N) | 1422.68 ± 342.35 | 1487.08 ± 382.58 | 1448.77 ± 394.21 | 1473.22 ± 358.60 | 0.922 | 0.564 |  |
| Peak VGRF2 (N) | 1331.89 ± 423.01 | 1291.96 ± 391.22 | 1270.15 ± 326.44 | 1313. 61 ± 358.74 | 0.277 | 0.362 |  |
| First cushion time (s) | 0.27 ± 0.09 | 0.35 ± 0.18 ^A^ | 0.29 ± 0.10 ^B^ | 0.27 ± 0.09 ^B^ | 0.058 | 0.006 ^#^ |  |
| Propulsion time (s) | 0.42 ± 0.23 | 0.39 ± 0.22 | 0.39 ± 0.21 | 0.37 ± 0.18 | 0.203 | 0.235 |  |
| Off-ground time (s) | 0.34 ± 0.11 | 0.34 ± 0.06 | 0.34 ± 0.08 | 0.33 ± 0.07 | 0.360 | 0.061 |  |
| Second cushion time (s) | 0.19 ± 0.09 | 0.19 ± 0.09 | 0.20 ± 0.09 | 0.19 ± 0.09 | 0.559 | 0.853 |  |

^#^, P-value <0.05.

^A^, Significant difference among the ACF38, ACF28, ACF18, and EVA groups.

^B^, Significant difference among the ACF38, ACF28, and ACF18 groups.

^C^, Significant difference among the ACF38 and ACF28 groups.

Values are expressed as mean±SD.

ACF, artificial cartilage foam; EVA, ethylene-vinyl acetate; Peak VGRF1, Peak vertical ground reaction force during the first jump; Peak VGRF2, Peak vertical ground reaction force during the second jump.
